# An international phase iii randomised trial on the efficacy of helium/oxygen during spontaneous breathing and intermittent non-invasive ventilation for severe exacerbations of chronic obstructive pulmonary disease (the E.C.H.O.^ICU^trial)

**DOI:** 10.1186/2197-425X-3-S1-A422

**Published:** 2015-10-01

**Authors:** P Jolliet, L Besbes, F Abroug, J Ben Kheli, M Besbes, J-M Arnal, F Daviaud, J-D Chiche, B Lortat-Jacob, J-L Diehl, N Lerolle, A Mercat, K Razazi, C Brun-Buisson, S Bertini, A Corrado, J Texereau, L Brochard

**Affiliations:** Intensive Care and Burn Unit - CHUV, Lausanne, Switzerland; Fattouma Bourguiba University Hospital, Ariana, Tunisia; Abderrahmen Mami Hospital, Monastir, Tunisia; Font-Pré Hospital, Toulon, France; Cochin Hospital, Paris, France; Georges Pompidou European Hospital, Paris, France; Angers University Hospital, Angers, France; Henri-Mondor Hospital, Paris, France; Careggi University Hospital, Florence, Italy; Air Liquide Santé International, Paris, France; St Michael's Hospital-University Toronto, Toronto, Canada

## Introduction

Due to its reduced density, Helium/Oxygen (He/O_2_) reduces the work of breathing, intrinsic PEEP and hypercapnia more than Air/O_2_ during non-invasive ventilation (NIV) in COPD exacerbations [1, 2]. Two prospective, randomized multicenter trials were inconclusive in showing a benefit of He/O_2_ NIV on outcome (intubation, mortality, length of stay (LOS) in ICU) but were potentially underpowered [3, 4].

## Objectives

To evaluate whether 72-hr continuous He/O_2_ during both spontaneous breathing and NIV is superior to Air/O_2_ in reducing NIV failure (intubation or mortality during ICU stay) in severe hypercapnic COPD exacerbations. Secondary outcomes included physiological parameters, duration of ventilation, ICU and hospital LOS, 6-month recurrence and rehospitalization rates.

## Methods

Prospective, randomized multicenter (16 centers in 6 countries) trial, comparing the two gas mixtures for a maximum of 72 hours. Hypothesis was that He/O_2_ would reduce intubation rate from 25% to 15%, resulting in a total sample size of 670 patients. Spontaneous breathing and NIV were applied with specific devices for He/O_2_. Same ventilator was used in both arms.

## Results

**Figure 1:**
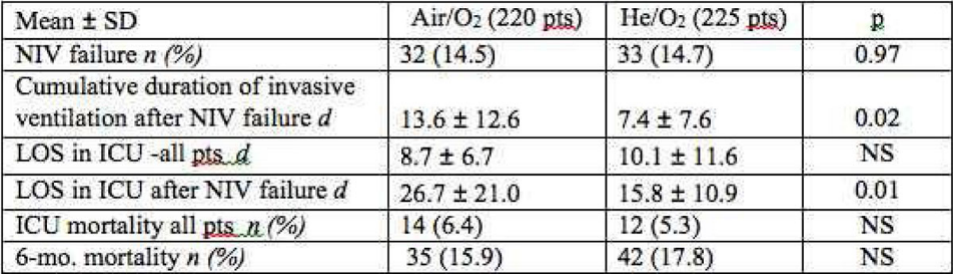
Figure 1

**Figure 2:**
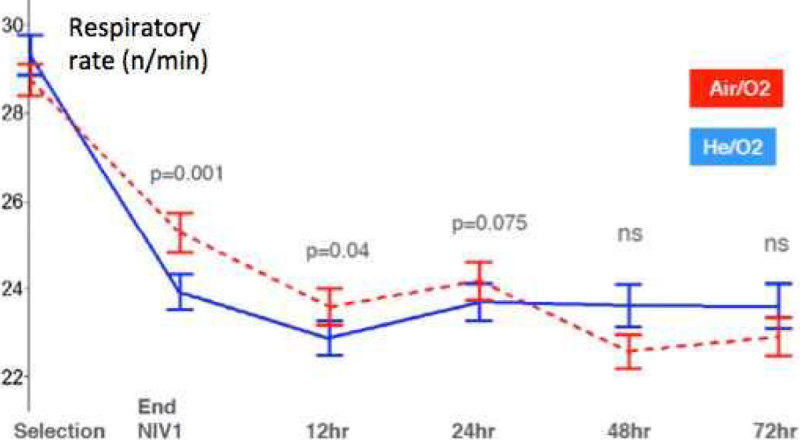
Figure 2

The trial was stopped prematurely for futility (low intubation rate reported by the adjudication committee). 445 patients were included (mean ± SD 68 ± 11 yrs; M:F 69:31%; BMI 26 ± 6 kg/m^2^; SAPS 3 49 ± 8; Resp rate (RR) 29 ± 6/min - PaO_2_ 75 ± 36 mmHg; PaCO_2_ 69 ± 16 mmHg; pH 7.30 ± 0.06 - intention-to-treat data set), with no baseline difference between He/O_2_ vs. Air/O_2_. The primary outcome was negative (Figure 1) and baseline pH was the only significant predictor of NIV failure. NIV failure occurred in the first 72 hours (while receiving the study treatment) in 58% of failures with He/O_2_ and 84% with Air/O_2_ (p = 0.97). RR (Figure 2), pH, PaCO_2_ and encephalopathy improved faster and with greater magnitude with He/O_2_

## Conclusions

NIV failure rate was not reduced by He/O_2_ administered during NIV and spontaneous breathing for up to 72 hrs. Failure rate was low in both groups, reflecting the current efficacy of NIV in decompensated COPD. However, He/O_2_ led to improved physiological response, thus confirming previous results, and a shorter duration of invasive ventilation and ICU stay in patients with NIV failure.

## Grant Acknowledgment

ClinicalTrials.gov Identifier: NCT01155310. Study funded by Air Liquide Healthcare.
